# The Application of a Piezo-Resistive Cardiorespiratory Sensor System in an Automobile Safety Belt

**DOI:** 10.3390/s150407742

**Published:** 2015-03-30

**Authors:** Syed Talha Ali Hamdani, Anura Fernando

**Affiliations:** 1 Textiles Research Group, School of Materials, The University of Manchester, Manchester, M13 9PL, UK; E-Mail: hamdani.talha@ntu.edu.pk; 2 Smart Materials Research Group, Department of Weaving, National Textile University, Sheikhupura Road, Faisalabad 37610, Pakistan

**Keywords:** woven, safety belt, cardiorespiratory signals, wearable sensors, piezo-resistive materials, ballistocardiography

## Abstract

Respiratory and heart failure are conditions that can occur with little warning and may also be difficult to predict. Therefore continuous monitoring of these bio-signals is advantageous for ensuring human health. The car safety belt is mainly designed to secure the occupants of the vehicle in the event of an accident. In the current research a prototype safety belt is developed, which is used to acquire respiratory and heart signals, under laboratory conditions. The current safety belt is constructed using a copper ink based nonwoven material, which works based on the piezo-resistive effect due to the pressure exerted on the sensor as a result of expansion of the thorax/abdomen area of the body for respiration and due to the principle of ballistocardiography (BCG) in heart signal sensing. In this research, the development of a theoretical model to qualitatively describe the piezo-resistive material is also presented in order to predict the relative change in the resistance of the piezo-resistive material due to the pressure applied.

## 1. Introduction

Electronic textiles (e-textiles) are helping existing and conventional electronics to improve their physical flexibility and integration of interconnections to the electronic interface. The technologies emerging in the e-textiles field are leading towards complete wearable sensing systems that could be an integral part of everyday outfits [1]. The increased interest of researchers and industrialists in the field of wearable sensors has caused the work done in this area to gain lot of importance in the last few decades [2]. Specifically, the capability to use these wearable sensors to ubiquitously monitor the health of patients over a long period of time has gained them much respect in the medical field. There, these wearable sensors are used to communicate real time patient medical feedback of possible forthcoming health threats to patient carers [3,4]. Due to the unpredictability of cardiorespiratory diseases, continuous monitoring of these bio-signals at early stages [5] is advantageous for ensuring human health [6]. Diseases like sleep apnoea syndrome and sudden infant death syndrome (sudden unexplained death before the age of one year) are conditions that are directly related to respiration and cardiac abnormalities respectively, requiring continuous and long term health monitoring [7,8]. When there is an abnormality in the function of the human body, the nervous system may cause an increase in the heart rate or respiration cycles. The blood pressure may also increase as well, along with many other abnormal indications. If such abnormalities occur repeatedly in the body, it can affect the performance of the cardiac system, which can eventually present itself as a heart attack [9]. The respiration and heart rate also reflect the emotions [10,11] and mental stresses [12] a person experiences [13]. One of the proactive approaches available to avoid such diseases and to look after the health in daily life is the ubiquitous monitoring of the human physiology.

This ubiquitous monitoring can be achieved by integrating biosensors into portable health monitoring devices. Due to the importance of automobiles in everyday life, the integration of biosensors into an automobile’s interior materials, namely the car safety belt or car seat cover can greatly strengthen the health monitoring action. According to World Bank statistics [14], more than 50% of the population in United Kingdom uses motor vehicles. Similarly, through surveys conducted in New South Wales, Australia, it was found that 69% of the general population generally drive to work [15], indicating the extent to which a bio-signal monitoring system in motor vehicles could influence the general health of the population.

In respiration monitoring, the spirometer is reputed to be the most accurate instrument available, as it is less sensitive to motion artefacts. However, the disadvantage of the spirometer is it’s incompatibility for integration with a non-invasive wearable system. Various studies have been conducted to capture cardiorespiratory signals using a wider range of wearable technologies [6,16,17,18,19,20,21,22]. The very first study in the field of wearable technology for capturing the cardiorespiratory signals was conducted by Gopalsamy *et al.* in 1999 [23]. The main purpose of that study was the transmission of heart signals from a wearable shirt integrated with solid state sensors. Other technologies for heart signal sensing involve technologies such as LED and photodiodes [24], microwave Doppler-ultrasonic techniques [25,26], optical fibers [27], piezoelectric [28] and piezo-resistive [29] materials. Out of these, piezo-resistive nonwoven fabrics can be used as active electrodes for the measurement of bio-potential and respiratory signals [30]. In contrast, in the current research, a nonwoven fabric impregnated with a piezo-resistive ink has been used as the cardiorespiratory sensor material integrated into an automobile safety belt.

In the current study a nonwoven cardiorespiratory sensor material was manufactured using a conductive particle filled polymer ink, generally known as an electrically conductive polymer composite (ECPC). For the optimum performance of the ECPC [31], the size of the conductive particles is an important factor since a large change of resistance can be observed when nanosize particles are used as compared to microsize particles [32]. Nanosize conductive particles are used to develop highly sensitive piezo-resistive materials having a gauge factor as high as 2900 [33]. However other parameters that have an impact on the sensitivity of piezo-resistive materials are their thickness, the ambient temperature [34] and the percolation concentration [35]. In review studies conducted by Lux [36] an attempt was carried out to find the factors affecting the percolation concentration. These factors include filler distribution, aspect ratio, filler/matrix interactions and the processing technique [34,37,38,39].

In addition, according to research conducted by Knite *et al.* [40], in order to obtain the best composition for sensor applications, the fiber volume fraction, fiber distribution and ratio between the fiber length and fiber diameter need to be considered too. Further investigations carried out by Pham *et al.* [41] and Hu *et al.* [42] revealed that the sensitivity of a nonwoven piezo-resistive sensor can be improved by improving the filler particle loading, degree of filler particle dispersion and sensor fabrication process.

## 2. Experimental Section

**Figure 1:**
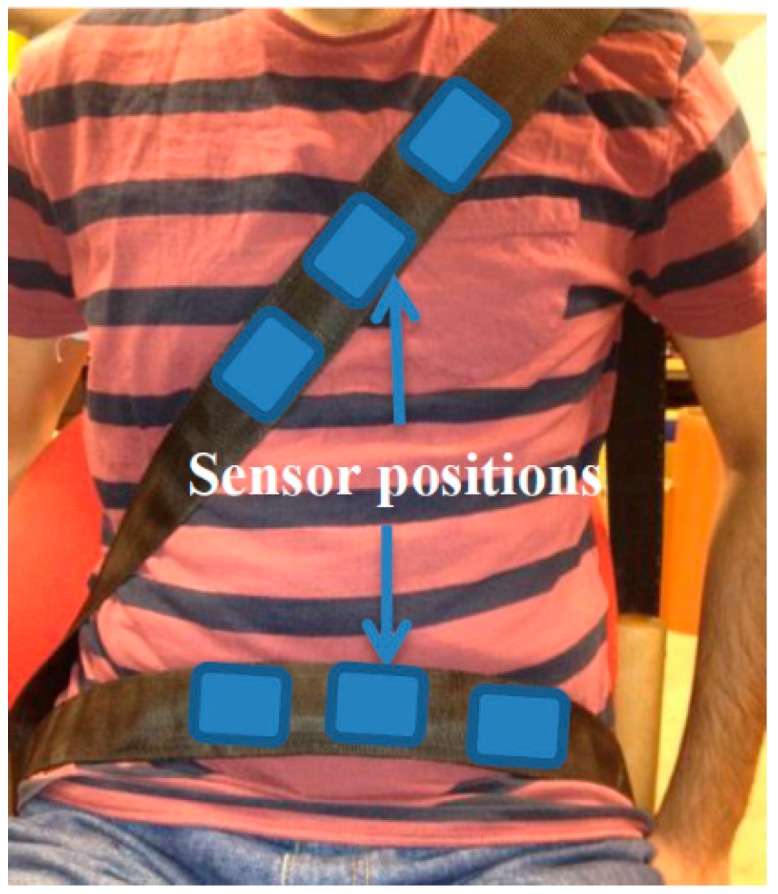
Subject wearing the prototype safety belt.

The construction of an automobile safety belt integrated with sensors mainly consists of three manufacturing stages: the safety belt weaving process, the preparation of sensors and their integration into the safety belt. The safety belt was woven using a high performance narrow fabric weaving machine where the piezo-resistive sensors were integrated into the safety belt as a post-construction process. The conductive paths, made of electro-conductive yarn too were also created on either side of the sensor during the weaving process to acquire the electrical signals from them. The sensors in the current case were constructed out of a nonwoven piezo-resistive material while electrodes made with silver knitted fabric were used to acquire the electrical signals resulting from the mechanical compression the sensor experiences. The knitted structure used in the knitted silver electrodes allows for elastic bidirectional movement in both the warp and weft directions of the fabric making the structure suitable for applications experiencing either unidirectional or bidirectional deformation. The thickness of the silver knitted electrodes, was found to be an important factor during the experiments since increased thickness in the sensor material showed that they tend to absorb the weak mechanical compression signals given out by BCG signals from the heart. Numbers of these sensors were integrated near the thorax and abdomen position of the prototype belt in order to ensure the capture of the best breathing and heart rate signals. The general positions of the sensors with respect to the subject wearing this smart safety belt are shown in Figure 1.

**Figure 2:**
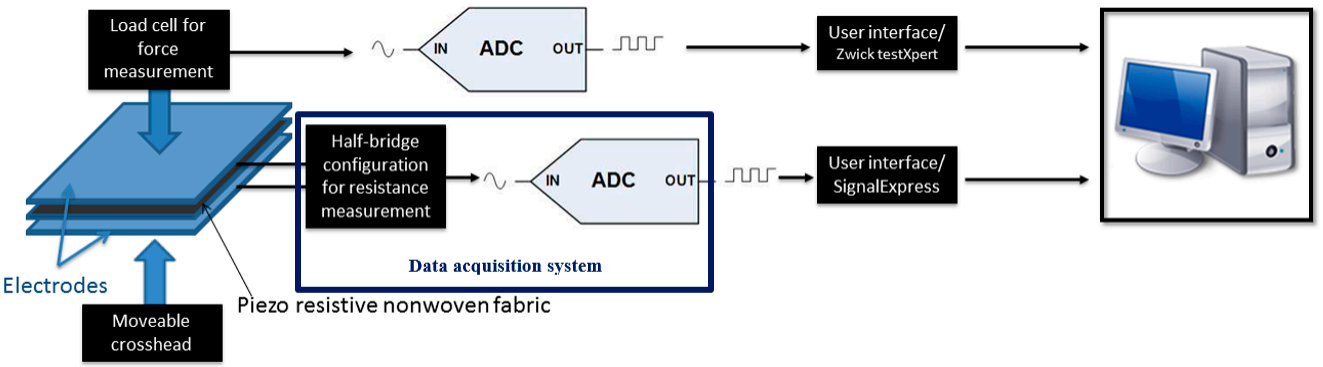
Experimental setup.

The characterization of sensors under mechanical load testing was carried out using the experimental setup shown in Figure 2. The cardio respiratory signals, in this case, were acquired from sensors integrated on safety belt using an NI-9219 data acquisition module.

## 3. Results and Discussion

**Figure 3:**
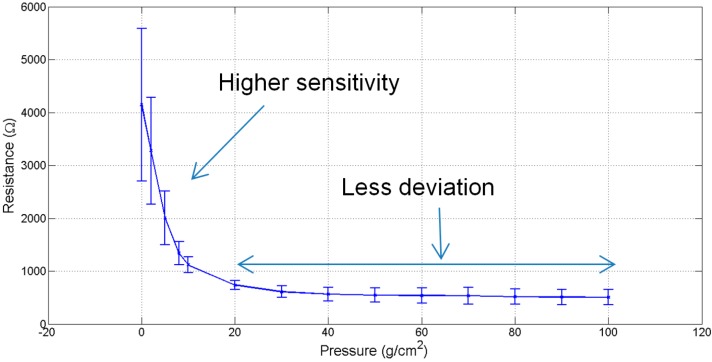
Sensors’ characteristic curve.

It can be seen from Figure 3 that the resistance of piezo-resistive sensors decreases sharply as the applied pressure is increased up to a critical value of 8 g/cm^2^. Thereafter beyond 20 g/cm^2^ the increase in pressure has a marginal effect on the resistance.

It was also observed that this sensor is responsive at very low pressure and capable of reacting to a weak signal due to a change in pressure. However due to the nature of the piezo-resistive nonwoven material, other than the peaks in the signal that correspond to cardiorespiratory activity, no characteristic signal or an absolute values for cardiorespiratory signals is acquirable. Additionally, the results are dependent on the posture of the subject, where proper placement of sensor and motion artefacts affects the quality of the signal. It is also to be noted that, for efficient mechanical compressive signal acquisition, the belt needs to be wrapped on a curved area on the body rather than a flat surface. This is because the belt wrapped on a convex shape is capable of reaching the critical pressure of 8 g/cm^2^.

### 3.1. Theoretical Modelling and Qualitative Validation of Sensor Characteristic Curve

**Figure 4:**
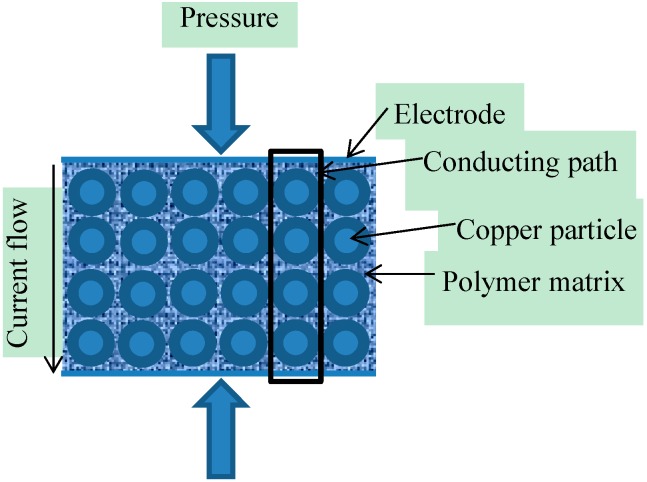
Schematic diagram of the piezo-resistive sensor.

In the current case, the theoretical model was created by using some of the work presented by Zhang *et al.* [43] and Knite *et al.*, [40]. In the current application the piezo-resistive sensor is created by sandwiching the aforementioned nonwoven piezo-resistive material between two silver electrodes. The theoretical derivation assumes that the uniform distribution of copper particles in the electro-conductive polymer matrix, as shown in Figure 4, results in uniformity of piezo-resistivity in the entire material. It was also assumed that the polymer matrix equally surrounds the circumference of the copper particles and thus results in an increase in their size. The electrodes are assumed to be in contact with the piezo-resistive material uniformly and the contact resistance between piezo-resistive material and electrodes is assumed to be negligible.

According to the quantum tunnelling theory, the tunnelling current of density ‘‘J’’ due to the material compression, at low voltage, created across the piezo-resistive material is given by the Equation (1) [43,44,45]:

(1) $$J=\frac{3\sqrt{2m_{e}\varphi }}{2s}.{\left(\frac{e}{h}\right)}^{2}.V.exp\left(-\frac{4\pi s}{h}\sqrt{2m_{e}\varphi }\right)$$

where:

J  tunnel current density;
$m_{e}$  electron mass;
φ  height of potential barrier;
s  inter particle separation;
e  electron charge;
h  Planck’s constant;
V  voltage supplied.

According to Sheng [46], the tunnel effect occurs only within a very small area of the surface; if the surface area is ‘‘$S_{a}$’’ and ‘‘V’’ is the supplied voltage then the resistance of the piezo-resistive material ‘‘$R_{pr}$’’ can be calculated using Equation (2).

(2) $$R_{pr}=\frac{V}{S_{a}\;J}$$

Substituting the value of ‘‘J’’ in Equation (2):

(3) $$R_{pr}=\frac{8\pi hs}{3S_{a}\gamma e^{2}}exp\left(\gamma s\right)$$

where;

(4) $$\gamma =\frac{4\pi }{h}\sqrt{2m_{e}\varphi }$$

The theoretical model for the characteristic resistive behavior of the complete sensor is given by Equation (5):

(5) $$\frac{R_{pr}}{R_{o}}=\frac{D.\left\{{\left(\frac{\pi }{6\theta }\right)}^{1/3}-1\right\}.\left(1-\frac{\sigma }{M}\right)}{D.\left\{{\left(\frac{\pi }{6\theta }\right)}^{1/3}-1\right\}}.exp\left[-\gamma \left\{D.\left\{{\left(\frac{\pi }{6\theta }\right)}^{1/3}-1\right\}.\left(1-\frac{\sigma }{M}\right)-D.\left\{{\left(\frac{\pi }{6\theta }\right)}^{1/3}-1\right\}\right\}\right]$$

The units of parameters in Equation (1) are given in Table 1.

**Table 1:** Parameters and their units used in Equation (1).

Symbol	Parameter	Units
D	Diameter of copper particle	nm
θ	Filler volume fraction	no units
σ	Stress applied on piezo-resistive material	g·cm^−2^
M	Compressive modulus	g·cm^−2^
γ	$\gamma =\frac{4\pi }{h}\sqrt{2m\emptyset }$	m^−1^

### 3.2. Calculation of Parameters

The compressive modulus ‘‘M’’ of the piezo-resistive material is calculated from the stress (σ) *vs.* strain (ε) curve for any two coordinates $\left({\text{ε}}_{1},{\text{σ}}_{1}\right)$ and $\left({\text{ε}}_{2},{\text{σ}}_{2}\right)$. The compressive modulus ‘‘M’’ of piezo-resistive material was calculated using Equation (6):

(6) $$\text{M}=\frac{{\text{σ}}_{2}-{\text{σ}}_{1}}{{\text{ε}}_{2}-{\text{ε}}_{1}}$$

The filler volume fraction of the piezo-resistive material depends on the number of layers, areal weight, filler density and the thickness of the piezo-resistive material. The areal weights of the copper particles and polymer matrix can be calculated by subtracting the areal weight of nonwoven fibers from the areal weight of the piezo-resistive material. The areal weight of copper particles and polymer matrix was found to be 3.45 × 10^−3^ g/cm^2^. The density of filler particles provided by the manufacturer [47] was $1.095\;\text{g}/{\text{cm}}^{3}$. The measured thickness of the piezo-resistive material was $6.36\times 10^{-2}\;\text{cm}$. For a single layer of piezo-resistive material (*n* = 1), the filler volume fraction ‘‘θ’’ can be calculated using Equation (7) [48].

(7) $$\text{Filler volume fraction }(\text{θ})=\;\frac{\text{n}.\text{W}}{\text{d}.\text{t}}$$

The height of the potential barrier for copper particle was calculated by Istratov *et al.*, [49,50] to be 0.55 eV. The numerical values for parameters and constants used to calculate the theoretical relative change in resistance of piezo-resistive material are given in Table 2.

**Table 2:** Required parameters and constants.

Parameter	Numerical Value
Electron mass (m)	9.10938291 × 10^−31^ kg
Filler volume fraction (θ)	4.96 × 10^−2^
Height of potential barrier between the adjacent particles (φ)	0.55 eV
Copper particle diameter (D)	50 nm

### 3.3. Validation of Results

**Figure 5:**
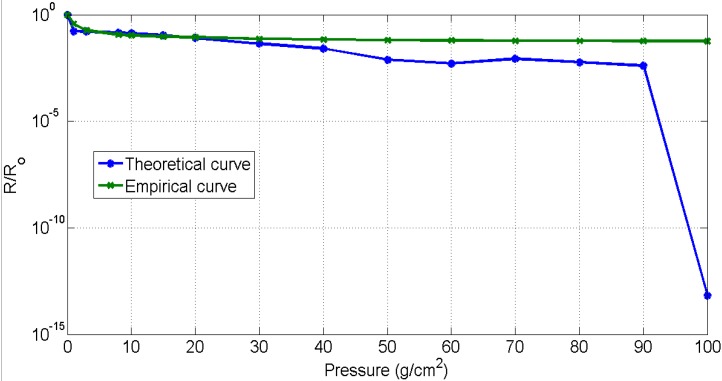
Results validation.

Figure 5 shows a comparison of theoretical and empirical curves. The trend of the theoretical “relative change in resistance” curve was observed to be similar to the empirical results. According to these curves, an exponential decrease in resistance was observed at low pressures. As explained in the works of Zang *et al.* [40] and Knite *et al.* [37], this decrease is due to the tunnelling effect, which the conductive particles experience while crossing the potential barrier. The main reasons for the decrease in resistance is directly due to the height of potential barrier and the interparticle separation in copper. Theoretically, no change in the resistance was observed when the copper particles cross the height of potential barrier and therefore the distance between the two adjacent particles become zero. Experimentally, it was found that the contact resistance between the electrodes and piezo-resistive material is higher at low pressure as compared to the resistance at high pressure. The main cause of contact resistance here is the air trapped between the electrodes and the piezo-resistive material. The decrease in contact resistance on the increase in pressure applied is due to the removal of air gaps between the electrodes and the piezo-resistive material. However, although the contact resistance decreases, it does not reach a null value. The deviation observed (Figure 5) between the theoretical curve and the empirical curve is due to this contact resistance.

### 3.4. Cardiorespiratory Signals

**Figure 6:**
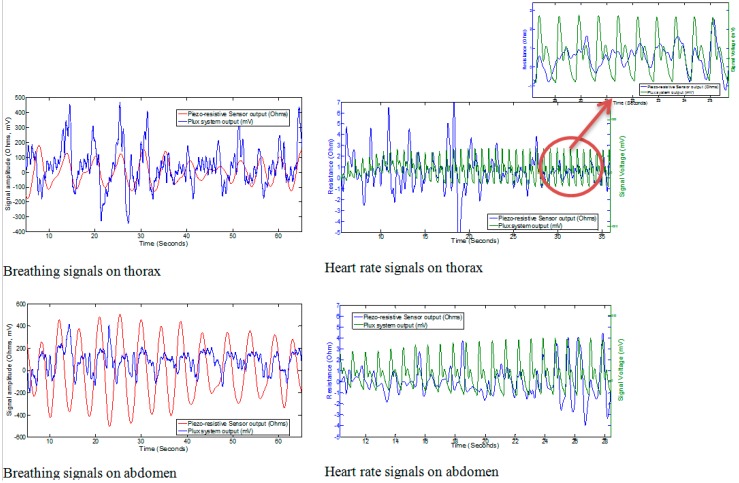
Results from prototype of safety belt.

The cardiorespiratory signals achieved from the current prototype safety belt are shown in Figure 6. It was observed that the sensor was capable of registering the respiration signals whereas the capturing of heart signal using this piezo-resistive sensor was quite challenging. Even after applying a band pass filter, it was found hard to differentiate between the noise and heart signals due to the similarity in the shape of both BCG signals and noise signals. In order to compare the heart signals captured using the piezo-resistive sensor with a BCG signal from a standard BCG signal capturing device, a commercially available BCG measuring system (biosignals plux, Lisbon, Portugal) was used. During the experiments, it was observed that the respiration signals obtained from the thorax and abdomen were quite smooth and the strength of the signal obtained from the sensor at abdomen was higher as compared to that of the sensor at thorax. This was mainly because of the larger volume of expansion of abdomen as compared to the volume of expansion of thorax.

In the case of capturing the heartbeat (BCG), the results achieved with the piezo-resistive sensor was similar to those presented by Paalasmaa and Ranta [51]; the ideal heart beat must not contain the same amplitude of every single beat, rather it should be enveloped in a bell-like form. The heartbeat was also observed to contain some additive noise. It was observed that instead of using signal filtering techniques, the heart beat from piezo-resistive sensors can also be acquired near the thorax position by holding the breath. However, as the impact of heartbeat is almost minimized near the abdomen it was not detected by the sensors placed at the abdomen. The summary of results obtained from the prototype of the safety belt is tabulated in Table 3.

**Table 3:** Strength of cardio respiration signals.

Position of Sensor	Breathing Signals (Ω)	Heart Signals (Ω)
Thorax	200–400	2~6
Abdomen	700–900	n/a

## 4. Conclusions

In the current research work, a piezo-resistive sensor showed quite promising results for capturing respiration signals. It was observed that stable respiration signals were achieved from the abdomen of test subjects due to the larger curved abdomen region as compared to the generally flat thorax. Capturing the heart signal using the current prototype sensor and without further advanced signal filtering techniques is challenging. However the heartbeat can be detected near the thorax position with a small signal amplitude, which is sufficient when advanced filtering is employed. Further research is being carried out to improve the sensitivity of the sensor and advanced filtering is being carried out to complement the sensitivity of the sensor in order to extract the heart rate.
